# Impact of dose reduction and iterative model reconstruction on multi-detector CT imaging of the brain in patients with suspected ischemic stroke

**DOI:** 10.1038/s41598-021-01162-0

**Published:** 2021-11-15

**Authors:** Karolin J. Paprottka, Karina Kupfer, Isabelle Riederer, Claus Zimmer, Meinrad Beer, Peter B. Noël, Thomas Baum, Jan S. Kirschke, Nico Sollmann

**Affiliations:** 1 Department of Diagnostic and Interventional Neuroradiology, School of Medicine, Klinikum rechts der Isar, Technical University of Munich, Munich, Germany; 2 TUM-Neuroimaging Center, Klinikum rechts der Isar, Technical University of Munich, Munich, Germany; 3 Department of Diagnostic and Interventional Radiology, University Hospital Ulm, Ulm, Germany; 4 Department of Radiology, Perelman School of Medicine, University of Pennsylvania, Philadelphia, PA USA; 5 Department of Radiology and Biomedical Imaging, University of California, San Francisco, CA USA

**Keywords:** Medical research, Stroke, Brain imaging, Medical imaging, Tomography, Computed tomography

## Abstract

Non-contrast cerebral computed tomography (CT) is frequently performed as a first-line diagnostic approach in patients with suspected ischemic stroke. The purpose of this study was to evaluate the performance of hybrid and model-based iterative image reconstruction for standard-dose (SD) and low-dose (LD) non-contrast cerebral imaging by multi-detector CT (MDCT). We retrospectively analyzed 131 patients with suspected ischemic stroke (mean age: 74.2 ± 14.3 years, 67 females) who underwent initial MDCT with a SD protocol (300 mAs) as well as follow-up MDCT after a maximum of 10 days with a LD protocol (200 mAs). Ischemic demarcation was detected in 26 patients for initial and in 64 patients for follow-up imaging, with diffusion-weighted magnetic resonance imaging (MRI) confirming ischemia in all of those patients. The non-contrast cerebral MDCT images were reconstructed using hybrid (Philips “iDose4”) and model-based iterative (Philips “IMR3”) reconstruction algorithms. Two readers assessed overall image quality, anatomic detail, differentiation of gray matter (GM)/white matter (WM), and conspicuity of ischemic demarcation, if any. Quantitative assessment included signal-to-noise ratio (SNR) and contrast-to-noise ratio (CNR) calculations for WM, GM, and demarcated areas. Ischemic demarcation was detected in all MDCT images of affected patients by both readers, irrespective of the reconstruction method used. For LD imaging, anatomic detail and GM/WM differentiation was significantly better when using the model-based iterative compared to the hybrid reconstruction method. Furthermore, CNR of GM/WM as well as the SNR of WM and GM of healthy brain tissue were significantly higher for LD images with model-based iterative reconstruction when compared to SD or LD images reconstructed with the hybrid algorithm. For patients with ischemic demarcation, there was a significant difference between images using hybrid versus model-based iterative reconstruction for CNR of ischemic/contralateral unaffected areas (mean ± standard deviation: SD_IMR: 4.4 ± 3.1, SD_iDose: 3.5 ± 2.3, *P* < 0.0001; LD_IMR: 4.6 ± 2.9, LD_iDose: 3.2 ± 2.1, *P* < 0.0001).  In conclusion, model-based iterative reconstruction provides higher CNR and SNR without significant loss of image quality for non-enhanced cerebral MDCT.

## Introduction

Non-contrast cerebral computed tomography (CT) is one of the most frequently performed radiological examinations and the first-line diagnostic approach for emergency evaluation of patients with suspected stroke^1–4^. It is recommended by the American Heart Association as the initial emergency modality for investigation^5^, which is mostly thanks to the high speed, wide availability, and feasibility of CT in most institutions.

Due to attenuation differences of healthy brain parenchyma, the absence of the gray matter (GM)/white matter (WM) interface is a well-known and notable early CT sign for delineation of the infarct in patients with ischemic stroke^6,7^. Yet, image noise in cerebral CT data is particularly problematic for assessment of this characteristic feature of ischemic stroke and can aggravate detection of infarcted areas^8–10^, because the difference in attenuation of normal brain tissue at the GM/WM boundary is as low as 5 to 10 Hounsfield Units (HU)^11^. Therefore, image noise has to be kept as low as possible in order to improve the visualization of the normal GM/WM interface.

As the photoelectric component for GM is roughly about 5% higher than the value of WM^12^, brain tissue represents therefore an optimal tissue for improving image quality by alternating dose parameters for CT acquisitions. Raising of the tube current during CT acquisitions results in an increase of the contrast-to-noise-ratio (CNR)^12^, which can also facilitate a reduction of image noise^13^. However, this would lead to an increased radiation exposure for the patient. Against the background of ever-increasing numbers of CT examinations and related cancer risk ratios, CT-based radiation exposure should be kept as low as possible to prevent harm to the patient^14–16^.

As another useful tool to improve image quality by reducing image noise, various CT scanner vendors have developed different image reconstruction algorithms as alternatives to the traditionally used filtered back projection (FBP)^17–20^. The concept of iterative reconstruction was first described decades ago. Until today, most commercially available algorithms are not fully iterative but use a combination of iterative reconstruction and a conventional reconstruction algorithm such as FBP, commonly referred to as hybrid iterative reconstruction^18–20^. Comparable to FBP, a backward projection step is used for such hybrid algorithms, but they are more advanced given that they can iteratively filter the raw data to reduce artifacts, and after the backward projection, the image data are iteratively filtered to reduce image noise^18–20^. A fully iterative method is more demanding, using raw data that are backward projected into the cross-sectional image space, which is followed by forward projection to compute artificial raw data^18–20^. Importantly, this data forward projection step is crucial to the algorithms, given that it establishes a physically correct modulation of the acquisition process^18–20^. Artificial raw data are then systematically compared to the initial true raw data to revise the cross-sectional images, while, simultaneously, a regularization step is implemented to remove image noise^18^. Backward and forward projections are repeated so that discrepancies between true and artificial raw data can be minimized^18–20^. With advancements in CT technique and increased computational power, fully iterative reconstruction methods become increasingly available. Although distinct technical details and names for reconstruction algorithms can vary between manufacturers, it is generally acknowledged that iterative model-based approaches provide images with improved noise and artifact reduction whilst requiring prolonged reconstruction speed, which should, however, not be a clinically relevant issue with modern CT systems^18^. Specifically, model-based iterative algorithms may help to increase the visibility of anatomical details of brain structures and the GM/WM interface. Consequently, these algorithms may have the potential to increase the sensitivity for detection of parenchymal brain lesions in ischemic stroke.

The aim of our study was to evaluate the impact of a model-based iterative image reconstruction algorithm for non-contrast cerebral multi-detector CT (MDCT) in patients with suspected ischemic stroke. We therefore compared the image quality and diagnostic value of scans with model-based iterative image reconstruction with those of scans using hybrid reconstruction considering MDCT acquisitions with standard dose (SD) and low dose (LD), respectively.

## Material and methods

### Study design and patient inclusion

All image acquisitions were performed at one institution and according to clinical indications, which were based on (1) the requirement for initial imaging due to suspected ischemic stroke, or (2) follow-up imaging in the context of a control scan after mechanical recanalization and/or thrombolytic therapy. Eligible patients who had both initial and follow-up imaging by non-contrast cerebral MDCT available at our department were identified in our hospital’s picture archiving and communication system (PACS).

Inclusion criteria were (1) MDCT according to the hospital-intern standard stroke protocol (non-contrast cerebral CT with SD, CT angiography of supraaortal and intracranial vessels, and CT perfusion), (2) follow-up cerebral MDCT (non-contrast cerebral CT with LD) on the same MDCT system, (3) follow-up cerebral magnetic resonance imaging (MRI) according to a hospital-intern standard stroke protocol (including diffusion-weighted imaging [DWI] sequences), and (4) diagnosis of cerebral ischemia or no intracranial pathology according to all available imaging data. The exclusion criteria were (1) interval between the initial and follow-up MDCT examination of more than 10 days, (2) incomplete coverage of the neurocranium or artifacts due to foreign bodies or motion, (3) age below 18 years, and (4) an intracranial pathology other than ischemia (e.g., bleeding or tumor). Overall, 131 patients were eligible and included in this study, with an interval of study enrollment from November 2018 to September 2020.

### Imaging by multi-detector computed tomography

Image acquisition was performed in supine position using a 128-slice MDCT scanner (Ingenuity Core 128, Philips Healthcare) in all patients. An initial scout scan was used for planning of the field of view (FOV), and subsequent helical scanning was acquired with implicit tube current modulation for non-enhanced cerebral MDCT examinations. Initial SD and follow-up LD scans were performed with a tube voltage of 120 kV, while the tube current was decreased in the LD protocol (343 mA versus 229 mA).

The datasets derived from SD and LD scanning were both reconstructed with an axial slice thickness of 5 mm using two different image reconstruction algorithms, which were provided by the vendor (hybrid algorithm: iDose4, iterative model-based algorithm: IMR3, Philips Healthcare). The distinct regularization level for the iterative model-based algorithm was determined for clinical routine scanning by a consensus decision (reached by six board-certified neuroradiologists) directly after implementation of this method at our institution (in 2018) and used consistently thereafter as a hospital-intern standard. The volumetric CT dose index (CTDIvol) and dose-length product (DLP) were extracted from the automatically generated dose reports. Table 1 provides an overview of scanning details for MDCT imaging.

**Table 1:** Scanning details and image reconstruction for scanning with standard dose (SD) and low dose (LD).

	Standard-dose (SD) imaging	Low-dose (LD) imaging
Scan increment (in mm)	10.0	
Cycle time (in s)	2.5	
No. of cycles	18	
Scan angle	420	
Rotation time	0.75	
Tube voltage (in kV)	120	120
Tube current (in mA)	343	229
Exposure (in mAs)	300	200
Volumetic CT dose index (in mGy)	46.6 ± 1.2 (range: 38.5–47.6)	31.2 ± 1.8 (range: 20.1–46.8)
Collimation width	16 × 0.625	
Slice thickness (axial, in mm)	5	
Image reconstruction	IMR3 and iDose4	
Windowing	Standard setting of window width of 80 HU and window length of 40 HU, individually adjustable	

### Qualitative image analysis

Qualitative image evaluation was performed using a standard PACS viewer (IDS7, Sectra AB). Two radiologists (reader 1 [R1], board-certified radiologist with 8 years of experience and reader 2 [R2], resident with 4 years of experience in stroke imaging) systematically assessed all imaging data in all patients. Evaluations were performed after patient pseudonymization, and the readers had no access to the clinical reports for original imaging as generated during clinical routine and were unaware of the distinct clinical indication that resulted in MDCT imaging.

All imaging data were assessed separately, with the readers being strictly blinded to the ratings of each other. Furthermore, the order of patient cases was randomized per reading round (four reading rounds: SD_IMR, LD_IMR, SD_iDose, and LD_iDose), with an interval of at least two weeks between single rounds to minimize recall bias. Overall image quality, anatomic detail, and differentiation of GM/WM were evaluated based on 5-point Likert scales for all datasets (Table 2). In case of ischemic demarcation, both readers rated the conspicuity of such demarcation on another 5-point Likert scale.

**Table 2:** Scoring scheme for qualitative image analysis.

Item	Score				
	1	2	3	4	5
Overall image quality	Poor	Fair	Medium	Good	Excellent
Anatomic detail					
GM/WM differentiation					
Conspicuity of ischemic demarcation					

### Quantitative image analysis

**Figure 1:**
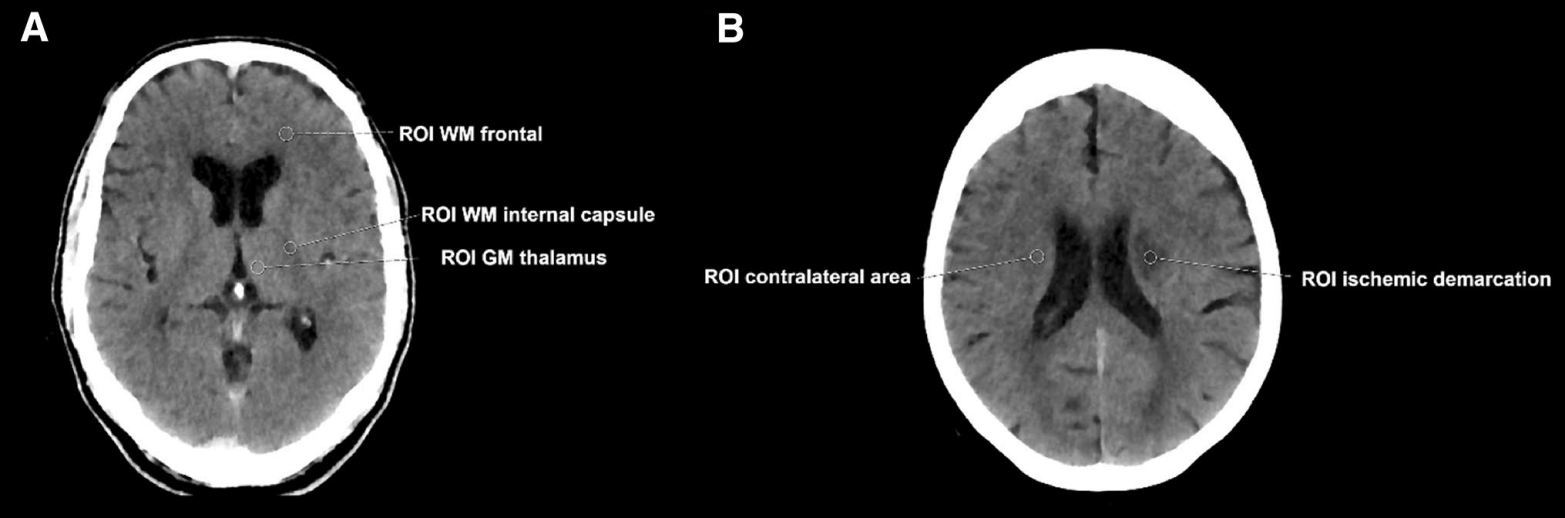
Placement of regions of interest (ROIs). (**A**) Placement of circular ROIs for the white matter (WM) of the left frontal lobe, WM of the left internal capsule, and gray matter (GM) of the left-sided thalamus (using axial slices at the level of the basal ganglia/third ventricle); (**B**) Placement of circular ROIs within ischemic demarcation (adjacent to the left lateral ventricle) and within a homologue, unaffected area of the contralateral hemisphere (using axial slices at the level of ischemic demarcation).

Similar to previous studies on CT scan quality assurance^21–24^, R1 used the following approach to perform quantitative image analysis. An axial slice at the level of the basal ganglia and third ventricle was chosen and measurements were taken in three regions of interest (ROIs) of identical size per patient (Fig. 1). One ROI was used to measure the attenuation (in HU) of WM in the left (or in case of ischemia unaffected) frontal lobe. Additional ROIs were placed to measure thalamic GM as well as WM of the posterior limb of the internal capsule on the left side (or, if affected by ischemic demarcation, on the unaffected right side). In case of ischemic demarcation another axial CT slice was chosen at the level of demarcation and two further ROIs were set: one in the core of the demarcated ischemic area and one within the same region of the unaffected contralateral hemisphere (Fig. 1).

Based on the values obtained, the signal-to-noise ratio (SNR) was calculated for the thalamus, the frontal WM, and the posterior limb of the internal capsule of the left or unaffected hemisphere using the following formula:

$${\text{SNR}} = { }\frac{{{\text{mean}}\;{\text{attenuation}}\;{\text{ROI}}}}{{{\text{StdDev}}\;{\text{of}}\;{\text{mean}}\;{\text{attenuation}}\;{\text{ROI}}}}$$

Additionally, the CNR was calculated for the GM/WM differentiation using the following formulas for all patients together as well as only for patients with ischemic demarcation:

$${\text{CNR}}_{{{\text{all}}\_{\text{patients}}}} = { }\frac{{{\text{mean}}\;{\text{attenuation}}\;{\text{ROI}}\;{\text{GM}}- {\text{mean}}\;{\text{attenuation}}\;{\text{ROI}}\;{\text{WM}}_{{{\text{frontal}}}} }}{{\left( {\frac{{{\text{StdDev}}\;{\text{of}}\;{\text{mean}}\;{\text{attenuation}}\;{\text{ROI}}\;{\text{GM}} + {\text{StdDev}}\;{\text{of}}\;{\text{mean}}\;{\text{attenuation}}\;{\text{ROI}}\;{\text{WM}}_{{{\text{frontal}}}} }}{2}} \right)}}{ }$$

$${\text{CNR}}_{{{\text{patients}}\_{\text{ischemia}}}} = { }\frac{{{\text{mean}}\;{\text{attenuation}}\;{\text{ROI}}\;{\text{within}}\;{\text{ischemic}}\;{\text{area}} - {\text{mean}}\;{\text{attenuation}}\;{\text{ROI}}\;{\text{contralateral}}}}{{\left( {\frac{{{\text{StdDev}}\;{\text{of}}\;{\text{mean}}\;{\text{attenuation}}\;{\text{ROI}}\;{\text{ischemic}}\;{\text{area}} + {\text{StdDev}}\;{\text{of}}\;{\text{mean}}\;{\text{attenuation}}\;{\text{ROI}}\;{\text{contralateral}}}}{2}} \right)}}$$

### Statistical data analysis

GraphPad Prism (version 6.0; GraphPad Software Inc.) and SPSS (version 25.0; IBM SPSS Statistics for Windows, IBM Corp.) were used for statistical data analyses. The level of statistical significance was set at *P* < 0.05.

For patient details, scanning parameters, dose characteristics, and values derived from quantitative and qualitative evaluations, descriptive statistics including mean ± standard deviation (StdDev), median, range, and absolute frequencies were calculated. Friedman tests were conducted between the SNR derived from SD_IMR, LD_IMR, SD_iDose, and LD_iDose data for GM as measured in the thalamus as well as for frontal WM and the internal capsule, respectively, followed by Dunn's multiple comparisons test as a post-hoc analysis. Similarly, Friedman tests were performed for the CNR of GM/WM between SD_IMR, LD_IMR, SD_iDose, and LD_iDose data for all included patients, again using Dunn's multiple comparisons test as a post-hoc test. In patients with detected ischemic demarcation, Wilcoxon matched-pairs signed rank tests were conducted for the CNR of unaffected/demarcated parenchyma for SD_IMR versus SD_iDose and for LD_IMR versus LD_iDose data, respectively.

Inter-reader agreements for qualitative evaluation regarding overall image quality, depiction of anatomic detail, and differentiation of GM/WM, considering all enrolled patients, and for conspicuity of demarcated ischemic parenchyma against healthy tissue only in patients with detected ischemic demarcation were assessed by weighted Cohen’s kappa (κ). Specifically, κ was calculated between the ratings of R1 and R2 for SD_IMR, LD_IMR, SD_iDose, and LD_iDose separately. Further, Wilcoxon matched-pairs signed rank tests were performed to compare scorings for SD_IMR versus SD_iDose and LD_IMR versus LD_iDose for each reader separately. Wilcoxon matched-pairs signed rank tests were also performed to investigate differences in the CTDIvol or the DLP between SD and LD images.

### Ethical approval

The study was approved by the ethics committee of the Faculty of Medicine of the Technical University of Munich and performed in accordance with the Declaration of Helsinki.

### Informed consent

The requirement for written informed consent was waived by the ethics committee of the Technical University of Munich due to the study’s retrospective design.

## Results

### Cohort characteristics

Data of 131 patients (mean age: 74.2 ± 14.3 years; age range: 26.8–95.6 years; 67 females) met our inclusion criteria. The mean interval between initial SD and follow-up LD imaging was 1.4 ± 1.7 days (range: 0–10 days), and the mean interval between initial SD MDCT and MRI was 2.3 ± 2.3 days (range: 2–13 days). Vessel occlusion in the CT angiography of initial MDCT examinations was identified in 80 patients, perfusion deficits according to CT perfusion were detected in 83 patients.

**Figure 2:**
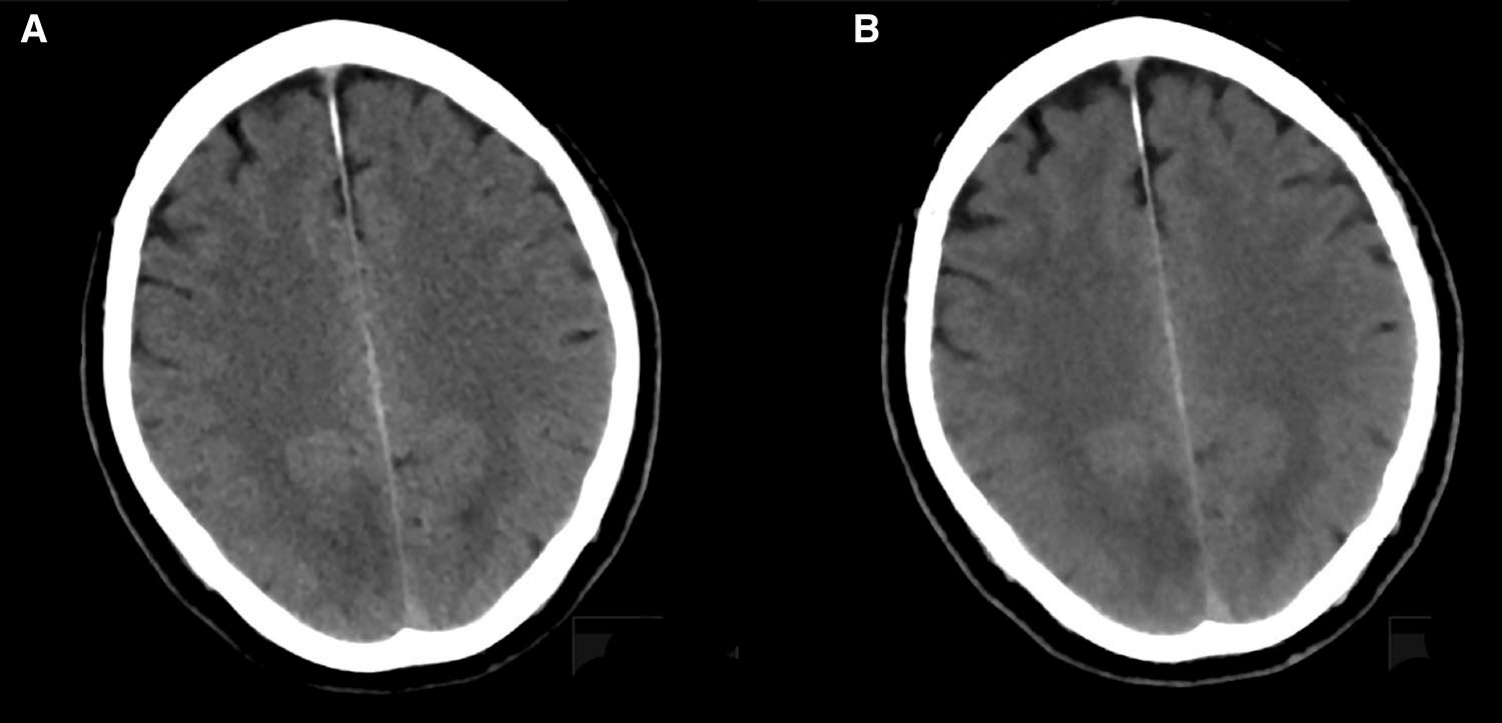
Exemplary patient case with ischemic demarcation (63-year-old male with visual disturbances). (**A**) Axial slices derived from scanning with low dose (LD) using a hybrid reconstruction algorithm (LD_iDose); (**B**) Corresponding axial slices from scanning with LD combined with a model-based iterative image reconstruction algorithm (LD_IMR). The demarcated area (parieto-occipital, right hemisphere) is more markedly depicted in (**B**), corresponding to a higher contrast-to-noise ratio (CNR).

Ischemic demarcation was detected initially in 26 patients, and it was present in 64 patients for follow-up LD imaging according to both readers (Fig. 2). Ischemic affection in these patients was confirmed by DWI sequences as derived from MRI. Characteristics of ischemia are shown in Table 3.

**Table 3:** Overview of detected ischemic stroke characteristics.

	Standard-dose (SD) imaging	Low-dose (LD) imaging
**Ischemic demarcation** (total number of patients)	26	64
Territory middle cerebral artery	18	27
Territory anterior cerebral artery	0	1
Territory posterior cerebral artery	2	4
Basal ganglia	0	13
Disseminated	0	1
Infratentorial	4	10
Multiple territories	2	8
**Side of demarcation**		
Right hemisphere	11	20
Left hemisphere	11	35
Bihemispheric	4	9

### Qualitative image analysis

According to qualitative evaluation, overall image quality was excellent on average for SD_IMR, LD_IMR, SD_iDose, and LD_iDose, respectively, with almost perfect inter-reader agreement (range of κ: 0.82–0.93) and without a statistically significant difference between SD_IMR versus SD_iDose for both readers and between LD_IMR versus LD_iDose for R1 only (*P* > 0.05; Table 4). Furthermore, high anatomic detail was depicted by all investigated data with substantial to almost perfect inter-reader agreement (range of κ: 0.61–0.89). Of note, while there was no statistically significant difference between SD_IMR versus SD_iDose (*P* > 0.05), the reconstruction algorithm had impact on anatomic detail for LD images, with statistically significantly better scores assigned by both readers for data reconstructed with IMR (*P* < 0.01; Table 4).

**Table 4:** Results of qualitative image evaluation from both readers (R1 and R2) using median and ranges for assigned scores.

	R1 (median, range)	R2 (median, range)	κ	p (SD_iDose vs. SD_IMR)		p (LD_iDose vs. LD_IMR)	
				R1	R2	R1	R2
**Overall image quality**							
SD_iDose	5 (3 – 5)	5 (3 – 5)	0.82	0.99	0.99	0.50	0.02
SD_IMR	5 (3 – 5)	5 (3 – 5)	0.82				
LD_iDose	5 (3 – 5)	5 (3 – 5)	0.86				
LD_IMR	5 (3 – 5)	5 (3 – 5)	0.93				
**Anatomic detail**							
SD_iDose	5 (3 – 5)	5 (4 – 5)	0.83	0.06	0.99	< 0.01	< 0.01
SD_IMR	5 (3 – 5)	5 (4 – 5)	0.61				
LD_iDose	5 (2 – 5)	5 (3 – 5)	0.89				
LD_IMR	5 (2 – 5)	5 (3 – 5)	0.86				
**GM/WM differentiation**							
SD_iDose	5 (4 – 5)	5 (4 – 5)	0.65	0.38	0.75	< 0.01	< 0.01
SD_IMR	5 (4 – 5)	5 (4 – 5)	0.44				
LD_iDose	5 (3 – 5)	5 (3 – 5)	0.89				
LD_IMR	5 (3 – 5)	5 (4 – 5)	0.79				
**Conspicuity of ischemic demarcation**							
SD_iDose	5 (3 – 5)	5 (4 – 5)	0.72	0.50	0.99	0.13	< 0.01
SD_IMR	5 (3 – 5)	5 (3 – 5)	0.96				
LD_iDose	5 (2 – 5)	5 (3 – 5)	0.87				
LD_IMR	5 (2 – 5)	5 (3 – 5)	0.86				

Images derived from scanning with standard dose (SD; SD_iDose & SD_IMR) and low dose (LD; LD_iDose & LD_IMR).

Very good differentiation between GM and WM was observed for all investigated data, with at least moderate to almost perfect inter-reader agreement (range of κ: 0.44–0.89). No statistically significant difference was observed between SD_IMR versus SD_iDose (*P* > 0.05) according to assessments of both readers, while statistically significantly better scores were obtained for LD data reconstructed with IMR when compared to iDose (*P* < 0.01; Table 4). In patients with demarcated ischemic parenchyma, conspicuity of ischemic demarcation was very good for all investigated data, and the agreement between scorings of both readers was substantial to almost perfect (range of κ: 0.72–0.96). Statistically significantly better conspicuity was observed for LD_IMR compared to LD_iDose according to evaluations of R2 (*P* < 0.01; Table 4).

### Quantitative image analysis

Regarding the SNR of GM as measured in the thalamus as well as for the SNR of frontal WM and the internal capsule, SD_IMR showed the highest values, respectively (mean ± StdDev: GM: 24.7 ± 7.9; WM frontal: 17.1 ± 4.2; WM internal capsule: 20.2 ± 7.5), followed by LD_IMR, SD_iDose, and LD_iDose (Table 5). Comparison of SNRs between SD_IMR, LD_IMR, SD_iDose, and LD_iDose for the SNRs measured in the different structures revealed a statistically significant difference (*P* < 0.01), yet the comparison between images using IMR was not significant according to post-hoc testing (Table 5).

**Table 5:** Results of quantitative image evaluation.

	Mean ± StdDev	Range	*P*	Dunn’s post-hoc test		
				Comparison	Rank sum diff	Sign
**SNR–GM thalamus**						
SD_iDose	20.6 ± 6.2	10.2–48.8	< 0.01	SD_iDose vs. SD_IMR	− 88.0	*
				SD_iDose vs. LD_iDose	116.0	*
SD_IMR	24.7 ± 7.9	11.9–64.3		SD_iDose vs. LD_IMR	− 78.0	*
				SD_IMR vs. LD_iDose	204.0	*
LD_iDose	16.1 ± 6.9	7.7–58.7		SD_IMR vs. LD_IMR	10.0	n.s
LD_IMR	23.3 ± 5.8	13.4–50.3		LD_iDose vs. LD_IMR	− 194.0	*
**SNR–WM frontal**						
SD_iDose	14.9 ± 5.3	6.7–54.0	< 0.01	SD_iDose vs. SD_IMR	− 104.0	*
				SD_iDose vs. LD_iDose	102.0	*
SD_IMR	17.1 ± 4.2	8.2–29.7		SD_iDose vs. LD_IMR	− 88.0	*
				SD_IMR vs. LD_iDose	206.0	*
LD_iDose	12.2 ± 4.1	5.1–35.8		SD_IMR vs. LD_IMR	16.0	n.s
LD_IMR	16.8 ± 4.5	4.9–27.9		LD_iDose vs. LD_IMR	− 190.0	*
**SNR–WM internal capsule**						
SD_iDose	16.9 ± 5.4	7.8–33.4	< 0.01	SD_iDose vs. SD_IMR	− 97.0	*
				SD_iDose vs. LD_iDose	116.0	*
SD_IMR	20.2 ± 7.5	3.7–73.3		SD_iDose vs. LD_IMR	− 57.0	*
				SD_IMR vs. LD_iDose	213.0	*
LD_iDose	13.3 ± 4.4	6.7–31.6		SD_IMR vs. LD_IMR	40.0	n.s
LD_IMR	18.6 ± 5.5	6.1–39.7		LD_iDose vs. LD_IMR	− 173.0	*
**CNR–GM/WM**						
SD_iDose	4.8 ± 1.6	0.8–10.6	< 0.01	SD_iDose vs. SD_IMR	− 105.0	*
				SD_iDose vs. LD_iDose	111.0	*
				SD_iDose vs. LD_IMR	− 84.0	*
SD_IMR	5.9 ± 2.0	1.4–15.7		SD_IMR vs. LD_iDose	216.0	*
LD_iDose	3.8 ± 1.4	0.5–10.6		SD_IMR vs. LD_IMR	21.0	n.s
LD_IMR	5.6 ± 1.9	1.2–11.5		LD_iDose vs. LD_IMR	− 195.0	*

Results of quantitative image evaluation using mean ± standard deviation (StdDev) for measurements. Images derived from scanning with standard dose (SD; SD_iDose & SD_IMR) and low dose (LD; LD_iDose & LD_IMR).

*n.s* not statistically significant.

*Statistically significant.

**Figure 3:**
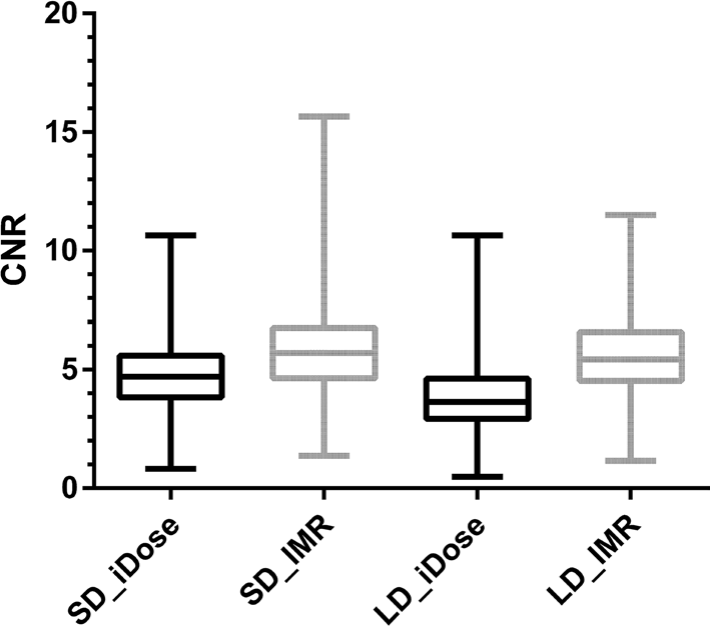
Contrast-to-noise ratio (CNR) of gray matter (GM)/white matter (WM) for all included patients. Box plots with minimum-to-maximum whiskers for the CNR of GM/WM using data from scanning with standard dose (SD; SD_iDose & SD_IMR) and low dose (LD; LD_iDose & LD_IMR).

**Figure 4:**
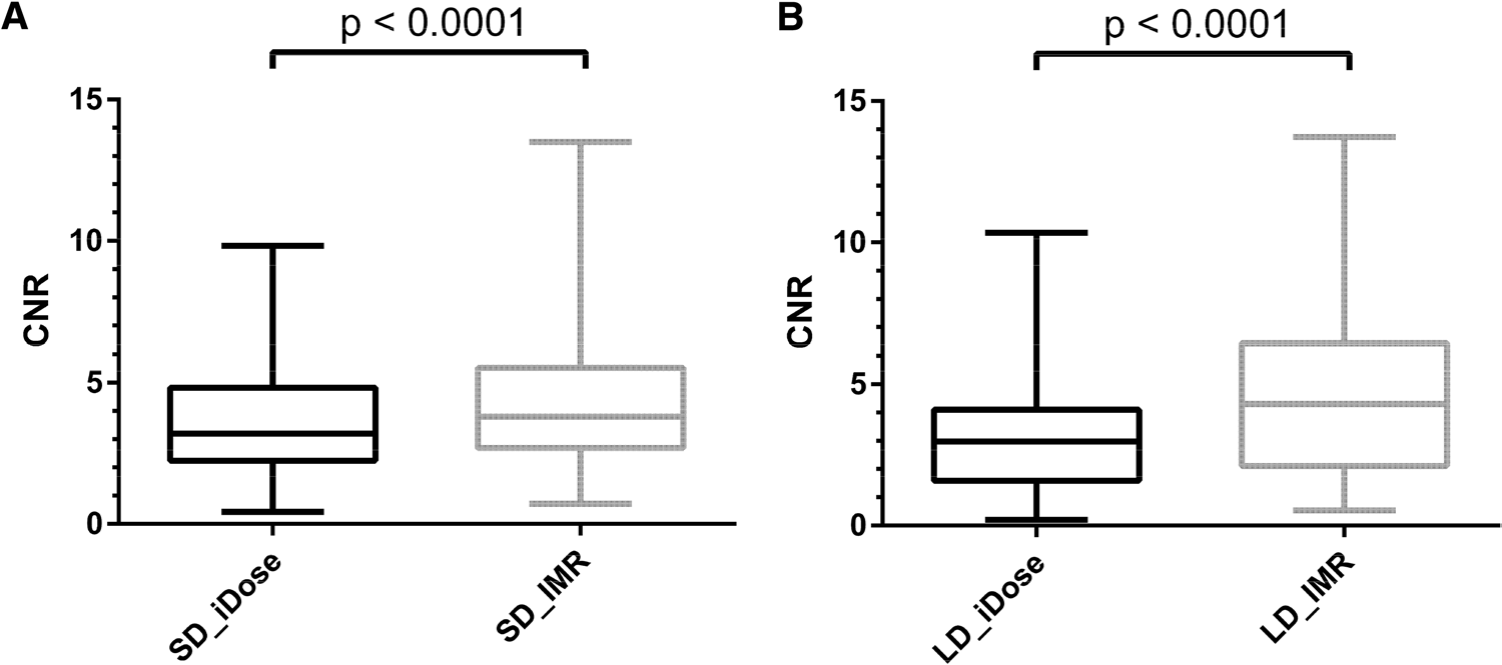
Contrast-to-noise ratio (CNR) in patients with ischemic demarcation. Box plots with minimum-to-maximum whiskers for the CNR ischemic demarcation/contralateral healthy parenchyma using data from scanning with standard dose (SD; SD_iDose & SD_IMR; **A**) and low dose (LD; LD_iDose & LD_IMR; **B**).

Similarly, for the CNR of GM/WM in all enrolled patients, highest values were obtained for SD_IMR (mean ± StdDev: 5.9 ± 2.0), followed by the results for LD_IMR, SD_iDose, and LD_iDose (Table 5; Fig. 3). The comparison of SD_IMR, LD_IMR, SD_iDose, and LD_iDose yielded a statistically significant difference (*P* < 0.01), however post-hoc testing of SD_IMR versus LD_IMR was not statistically significant (Table 5). For patients with ischemic stroke and detected ischemic demarcation, there was a statistically significant difference between images using iDose and IMR for both SD imaging (mean ± StdDev: SD_IMR: 4.4 ± 3.1; SD_iDose: 3.5 ± 2.3; *P* < 0.0001) and LD imaging (mean ± StdDev: LD_IMR: 4.6 ± 2.9; LD_iDose: 3.2 ± 2.1; *P* < 0.0001), with application of IMR leading to significantly higher CNR (Fig. 4).

### Radiation dose

The mean CTDIvol for SD images amounted to 46.6 ± 1.2 mGy (range: 38.5–47.6 mGy), and it was 31.2 ± 1.8 mGy (range: 20.1–46.8 mGy) for LD images. Correspondingly, the mean DLP amounted to 673.6 ± 48.6 mGy*cm (range: 571.0–857.5 mGy*cm) for SD data and 441.9 ± 33.0 mGy*cm (range: 381.0–666.3 mGy*cm) for LD images. The difference between SD and LD data was statistically significant for the CTDIvol and the DLP, respectively (*P* < 0.01).

## Discussion

Our results indicate higher SNR and CNR for SD as well as LD imaging by non-contrast cerebral MDCT when a model-based iterative algorithm is used for image reconstruction, as compared to a hybrid reconstruction algorithm. This was observed for both unaffected brain parenchyma as well as for demarcated areas due to ischemia in initial SD and follow-up LD imaging, respectively. More specifically, a model-based iterative image reconstruction algorithm could provide better anatomic detail, GM/WM differentiation, and conspicuity of ischemic demarcation for non-enhanced cerebral MDCT using LD imaging. Except for evaluations of GM/WM differentiation using MDCT data acquired with SD and reconstructed with the model-based iterative algorithm, inter-reader agreement was substantial to almost perfect.

Head CT has most commonly been performed at a tube voltage of 120 to 140 kVp^25^. Up to now, the American Association of Physicists in Medicine recommends a peak tube voltage in conventional cerebral CT studies of 120 to 140 kVp, depending on the manufacturer as well as on the system^27^. In accordance with this recommendation, we performed our non-contrast cerebral MDCT scans with a tube voltage of 120 kV while reducing tube currents from 343 mA for SD to 229 mA for LD imaging (exposure of 300 mAs versus 200 mAs). To the best of our knowledge, no studies with comparable low tube currents have been performed in-vivo for cerebral non-enhanced MDCT considering patients with suspected ischemic stroke. In this regard, previous studies on the matter have demonstrated reductions of exposure to values in the range of about 350 mAs to 260 mAs^28,29^. The range of potential relative dose reduction for head CT is thus similar to the range reported throughout the body: paranasal sinus^30^, chest^31^, coronary arteries^32^, and abdomen^33^. As a result of tube current reduction, the image quality was lower for LD compared to SD imaging, but still showed mostly sufficient values for LD MDCT according to our assessments.

The application of fully iterative reconstruction approaches has potential to compensate for increases in image noise and artifacts with tube current reductions to a certain degree^18–20^. Bodelle et al. compared cranial CT scans of 51 patients with infarction performed with either a LD (260 mAs; n = 21) or SD (340 mAs; n = 30) protocol, which were reconstructed with a hybrid reconstruction algorithm as well as FBP considering, amongst other items, the conspicuity of infarcted areas^34^. They concluded that hybrid reconstruction makes possible a dose reduction (− 24%) without relevant constraints regarding imaging of the demarcation of ischemic lesions^34^. Results seem to correspond to those of Bricout et al., who showed that a LD protocol (using a hybrid reconstruction algorithm) enables a significant reduction of radiation dose without relevant image quality impairment as overall image quality was judged as good or excellent in patients with a suspicion of delayed cerebral ischemia after aneurysmal subarachnoid hemorrhage^28^. Ben-David et al. investigated the effect of dose reduction in non-contrast cerebral CT scans with regard to GM/WM contrast by reducing tube voltage from 120 to 80 kV^25^. As in our study they compared two CT scans with different doses acquired for the same patients at two different time points and assessed attenuation, noise, and CNR for different ROIs, concluding that the CNR of GM/WM per dose is increased by 40%^25^.

In general, model-based iterative reconstruction algorithms seem to offer higher noise reduction than previously used reconstruction methods^18,35^. For cerebral CT, this is proposed by two studies performed by Inoue and colleagues, who investigated the impact of model-based iterative reconstruction on the accuracy of stroke diagnosis for the posterior fossa and the territory of the middle cerebral artery, comparing 5 mm axial slices of cerebral CT reconstructed with FBP or model-based iterative reconstruction with regard to image noise and CNR^36,37^. The authors concluded that model-based iterative reconstruction provides a better diagnostic performance as well as a better image quality and improved hypo-attenuation detection in patients with acute stroke as image noise was significantly lower and the difference in CNR between the infarcted and non-infarcted areas was significantly higher for the model-based iterative reconstructions^36,37^. Their results are in accordance with those presented by Iyama et al., who also compared FBP and model-based reconstructions for cerebral CT^38^. They postulated that model-based reconstruction may improve not only the image quality but also the performance for the detection of parenchymal hypo-attenuation in patients with acute ischemic stroke^38^. While these studies investigated previously used FBP but not a more recently applied hybrid approach, Lombardi et al. compared the diagnostic value of a model-based iterative reconstruction algorithm with that of a hybrid algorithm for identifying the hyperdense artery sign as one of the earliest signs of ischemic stroke on non-enhanced CT^39^. The authors found that a model-based iterative approach significantly increased sensitivity in detecting a hyperdense artery sign, offering higher SNR and CNR in comparison with hybrid reconstruction algorithms^39^. Furthermore, Liu et al. evaluated the image quality and lacunar lesion detection of thin-slice head CT images with three different reconstruction algorithms (FBP, hybrid reconstruction, and iterative model-based reconstruction) by comparing routine images with FBP to those with hybrid and iterative model-based reconstructions, analyzing CT attenuation using CNR and noise measurements, an artifact index of the posterior cranial fossa, and subjective analysis of overall image quality^40^. They concluded that iterative model-based reconstruction can lead to better image quality^40^. However, their study excluded patients with ischemic stroke (except for lacunar infarcts), and they did not specifically investigate the impact of tube current reduction in combination with an iterative model-based reconstruction algorithm^40^. Hence, to date we are not aware of another study that compared hybrid versus model-based iterative image reconstruction for non-enhanced cerebral CT in patients with suspected acute stroke and ischemic demarcation. Thus, the results of the present study may provide relevant evidence for significantly improved image quality when using a model-based iterative image reconstruction approach for this very common use case in clinical routine. On the long run, this may potentially allow to decrease the radiation exposure during MDCT scanning even further, with aggravated image noise having greater chances to be compensated for by a model-based iterative approach.

Even though the differences regarding imaging quality and conspicuity of ischemic areas are minor, the inter-reader agreement in the blinded rating of both raters was substantial to almost perfect for most evaluated items and scans, except for evaluations of GM/WM differentiation using SD imaging data with model-based iterative reconstruction. In this regard, previous research has already suggested that the performance of human readers for assessing ischemic demarcation can depend on the algorithm used for MDCT image reconstruction, with a trend towards better agreement for more established reconstruction algorithms (i.e., hybrid algorithms) with the experience of the reader^41^. Thus, a comparable result may be present for ratings of GM/WM differentiation in SD imaging data with model-based iterative reconstruction, which might be interpreted as an analogous trend to higher variation between readers for the more recently introduced model-based iterative image reconstruction algorithm over the more established hybrid algorithm.

There are some limitations to our study. First, this was a retrospective study, and experienced readers might be able to detect whether model-based iterative or hybrid reconstruction was used for image reconstruction in selected cases. However, subjective qualitative and objective quantitative results seem in agreement, supporting potential benefits of iterative model-based reconstructions particularly for LD data. Second, this study only used tube current reduction with two levels for radiation dose reduction and a fixed level of regularization, which was based on a consensus decision at the time of introduction of iterative model-based reconstruction at our institution. Yet, the reconstruction parameters of iterative model-based algorithms can be tuned to improve visibility of objects with a low contrast and to further decrease image noise (e.g., by using other or multiple levels of regularization related to the clinical indication for imaging)^18^. Other approaches such as sparse sampling may be performed in the future on cerebral MDCT data to further exploit possibilities of further radiation dose restrictions. Yet, to date, potential benefits of this technique have been shown for other applications or body regions than non-enhanced cerebral MDCT^42–45^. Third, for ethical reasons, we could not perform a paired study with one patient undergoing two cerebral MDCT exams with different doses at the same time point. Yet, phantom studies that can apply multiple settings within the same scanning session could follow up on the results of this study. Fourth, for the LD examination that was performed up to 10 days after the initial SD exam, any ischemic area would naturally appear with clearer demarcation and would therefore be easier to detect than in the first hours after symptom onset. Importantly, in this study we did not directly compare initial SD to follow-up LD imaging for demarcated areas to avoid bias due to aggravated demarcation over time.

## Conclusion

A model-based iterative image reconstruction algorithm could provide better anatomic detail, GM/WM differentiation, and conspicuity of ischemic demarcation for non-contrast cerebral MDCT using a LD imaging protocol. On a similar note, the CNR of ischemic demarcation/contralateral healthy parenchyma could be improved by model-based iterative image reconstruction. Future studies including advanced acquisition schemes (e.g., sparse sampling) or other approaches for image reconstruction (e.g., fine-tuned iterative model-based reconstructions by using different dedicated regularization levels, or artificial intelligence-based image reconstruction algorithms) could facilitate additional decreases in radiation exposure without clinically relevant impact on image quality and diagnostic use.

## Abbreviations

CNR: Contrast-to-noise ratio
CT: Computed tomography
CTDIvol: Volumetric CT dose index
DLP: Dose-length product
DWI: Diffusion-weighted imaging
FBP: Filtered back projection
FOV: Field of view
GM: Gray matter
HU: Hounsfield Units
κ: Cohen’s kappa
LD: Low dose
MDCT: Multi-detector CT
MRI: Magnetic resonance imaging
PACS: Picture archiving and communication system
R1: Reader 1
R2: Reader 2
ROI: Region of interest
SD: Standard dose
SNR: Signal-to-noise ratio
StdDev: Standard deviation
WM: White matter
